# Silica Biomineralization with Lignin Involves Si–O–C Bonds That Stabilize Radicals

**DOI:** 10.1021/acs.biomac.4c00061

**Published:** 2024-05-07

**Authors:** Srinath Palakurthy, Lothar Houben, Michael Elbaum, Rivka Elbaum

**Affiliations:** † The Robert H. Smith Institute of Plant Sciences and Genetics in Agriculture, The Hebrew University of Jerusalem, 7610001 Rehovot, Israel; ‡ The Weizmann Institute of Science, 7610001 Rehovot, Israel

## Abstract

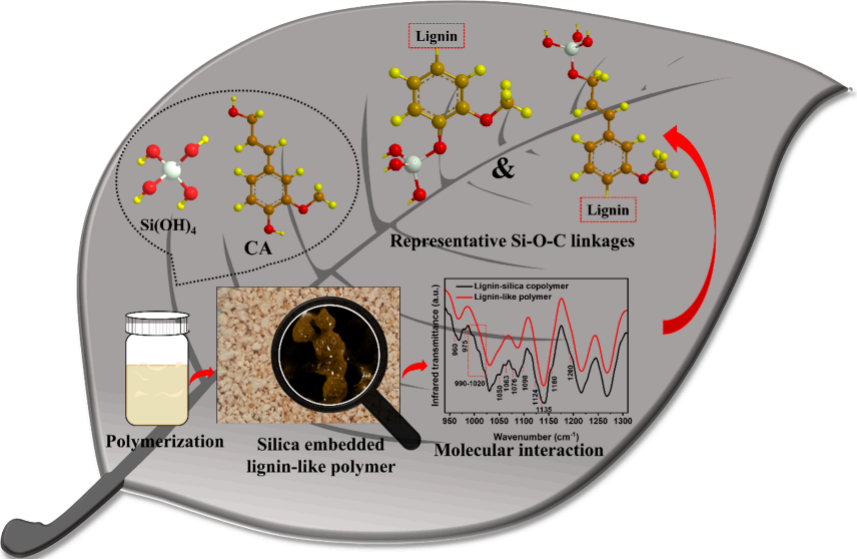

Plants undergo substantial biomineralization of silicon, which is deposited primarily in cell walls as amorphous silica. The mineral formation could be moderated by the structure and chemistry of lignin, a polyphenol polymer that is a major constituent of the secondary cell wall. However, the reactions between lignin and silica have not yet been well elucidated. Here, we investigate silica deposition onto a lignin model compound. Polyphenyl propanoid was synthesized from coniferyl alcohol by oxidative coupling with peroxidase in the presence of acidic tetramethyl orthosilicate, a silicic acid precursor. Raman, Fourier transform infrared, and X-ray photoelectron spectroscopies detected changes in lignin formation in the presence of silicic acid. Bonds between the Si–O/Si–OH residues and phenoxyl radicals and lignin functional groups formed during the first 3 h of the reaction, while silica continued to form over 3 days. Thermal gravimetric analysis indicated that lignin yields increased in the presence of silicic acid, possibly via the stabilization of phenolic radicals. This, in turn, resulted in shorter stretches of the lignin polymer. Silica deposition initiated within a lignin matrix via the formation of covalent Si–O–C bonds. The silica nucleants grew into 2–5 nm particles, as observed via scanning transmission electron microscopy and energy-dispersive X-ray spectroscopy. Additional silica precipitated into an extended gel. Collectively, our results demonstrate a reciprocal relation by which lignin polymerization catalyzes the formation of silica, and at the same time silicic acid enhances lignin polymerization and yield.

## 1 Introduction

Silicon (Si) and oxygen are the two most abundant elements of the earth’s crust, occurring naturally as SiO_2_ or silicates. Silicon oxide is absorbed as silicic acid (Si(OH)_4_) into a wide variety of organisms, including plants, and is then deposited in a process termed biosilicification to produce amorphous hydrated silica structures (SiO_2_·*n*H_2_O).^1^ Biological macromolecules under genetic control set the localization, formation rate, and physicochemical properties of the biomineral. Molecular biology and analytical techniques could be utilized to investigate the molecular machinery involved in the biomineral formation for the synthesis and applications of various materials.^2−4^

Plants absorb silicic acid via their roots, which is distributed with the water stream and eventually deposited as silica in cell walls and cell lumens. Silica content per dry weight may reach 0.2–10%, thus playing a major role in the plant structure. Interestingly, fertilizing plants with silica is correlated to increased yield, tolerance to pathogens and herbivores, metallic toxins, and other situations related to oxidative stresses.^5,6^ However, silica activity in reducing stress is poorly understood.^7^

The formation of silica within plants occurs under ambient temperature and pressure at neutral pH. Silicic acid can be concentrated by the concerted activity of silicon transporters,^8,9^ making plant metabolism an important factor in silicification.^10^ In some cases, the deposition of silica is controlled by biologically produced molecules.^11^ In particular, we showed that lignin may nucleate the formation of silica in sorghum roots.^12^ Lignin, as a cell wall component, acts as a template to produce biosilica.^13,14^ Silica forms in the presence of polymerized lignin but not its monomeric precursors, suggesting that silicic acid condensation is catalyzed by polymerized lignin and/or short lignin oligomers.^14,15^ The distribution and possible chemical bonds between silica and lignin have not been well described. This could be due to the complex structures and varied compositions of natural lignin. Addition of silica to lignin may have desired technological capabilities such as improved adsorption capacity of lignin for various pollutants in wastewater.^16^ The carbonaceous material derived from lignin with inorganic interfaces of silica exhibits huge potential in the electrochemical industry due to its high theoretical electrical capacity. For example, energy-related applications of lignin–silica hybrids are developed in the field of anode manufacturing of lithium-ion batteries.^17^

**Figure 1:**
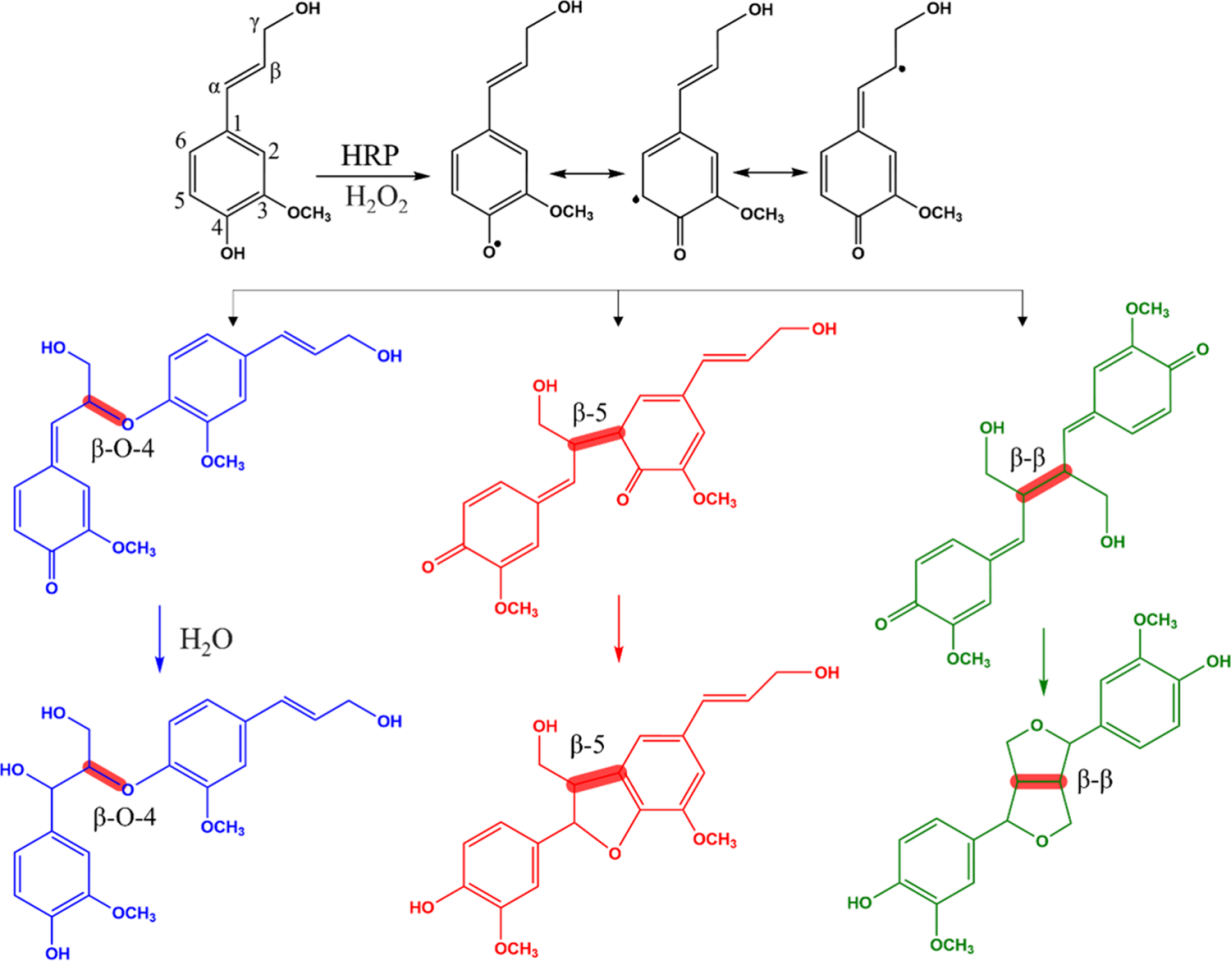
Major chemical species during radical coupling of coniferyl alcohol. Top: coniferyl alcohol dehydrogenation by hydrogen peroxide and horseradish peroxidase (HRP) leads to the formation of phenoxyl radicals. Middle: intermediate quinone methide species. Bottom: the main interunit linkages marked in red, β-O-4, β-5, and β–β.^18,19^

*In vitro* dehydrogenative polymerization of monolignols has been used widely in plant phenolic research as a model to study lignification. A common model for dehydrogenative polymerization is based on coniferyl alcohol, one of the most abundant lignin monomers.^18^ During polymerization, a coniferyl alcohol molecule is oxidized at the phenolic hydroxyl group, yielding a resonance-stabilized phenoxy radical (Figure 1). The free radical commonly undergoes coupling to another radical at the β-O-4 and β-5 positions, leading to a linear polymer. Branching may occur through a nucleophilic attack by water on the benzyl carbon of the unstable quinone methide intermediate and the formation of β–β coupling. However, this is less frequent under buffered pH.^18,19^

In this work, we aimed to obtain new insights into plant cell wall synthesis by understanding the chemistry between lignin and silica during their synthesis. To this end, a lignin-like polymer was produced in vitro through oxidative coupling polymerization of coniferyl alcohol catalyzed by HRP, and various concentrations of silicic acid were added to the polymerization reaction. Most significantly, we found that silyl ethers (Si–O–C) formed between lignin and silica. The polymerizing silicic acid increased the net polymerization of the lignin monomers. Si–O–C bonds limited silicic acid polymerization to short cyclic siloxanes. At the same time, they also suppressed lignin linear polymerization such that β–β coupling and branching were more common.

## 2 Materials and Methods

### 2.1 Oxidative Coupling Polymerization Process

Oxidative coupling reactions were performed according to a previous report that focused on the ability of lignin to catalyze silica formation.^15^ Coniferyl alcohol (3 mg, Sigma-Aldrich) dissolved in 100 μL of acetone was gently mixed with 3.7 mL of 100 mM potassium phosphate buffer (pH 7.4) containing horseradish peroxidase (HRP, 20 units equal to 20 μL of 5 mg/mL, Sigma-Aldrich) at 25 °C. Hydrogen peroxide solution (4 μL, 30% w/w) was added to the solution. The reaction mixture was then agitated at 60 rpm for 3 h or 3 days in the darkness. Centrifugation was performed using a Thermo Scientific Heraeus Pico 17 microcentrifuge at 5000 rpm (1400 g). The precipitates were rinsed three to four times with double-distilled water and dried in a desiccator under vacuum at room temperature. To produce the lignin–silica composite, 1 M silicic acid was produced by adding 150 μL of tetramethyl orthosilicate (TMOS, Sigma-Aldrich) to 850 μL of 1 mM HCl and mixing for 30 min. Silicic acid at final concentrations of 5, 10, 20, 35, 50, and 100 mM was added to the reaction solution before the addition of hydrogen peroxide.

### 2.2 Characterization

Raman spectra were recorded using a confocal Raman microscope (Renishaw, UK) equipped with a 532 nm laser of 45 mW power. Less than 0.5 mg of each compound in distilled water was mounted on a standard microscopy glass slide and covered with coverslips for measurements using a 63× water immersion objective. Spectra were collected with 100% laser intensity, 0.1 s acquisition time, and 10 accumulations per spectrum and analyzed using WIRE3.4 software.

X-ray photoelectron spectroscopy (XPS) narrow scan spectra were acquired by using an AXIS Supra (Kratos, Kratos Analytical Ltd.) instrument equipped with a high-resolution monochromatic Al Kα X-ray source. The samples were excited by Al Kα X-rays (*h*ν = 1486.7 eV) with a working voltage of 15 kV and emission current of 40 mA. High-resolution spectra of the O 1s and Si 2p energies were obtained in all samples. The spectra were then analyzed by using the built-in Kratos software (Version 1.5).

Fourier transform infrared (FTIR) spectra of the specimens were collected on a Nicolet 6700 spectrometer. Samples were mixed with KBr (Sigma) and pressed using a pellet presser to obtain transparent pellets of about 0.5 mm in thickness. Transmittance spectra were obtained between 400 and 4000 cm^–1^ with a resolution of 4 cm^–1^. Absorbance was calculated as a logarithmic function of transmittance (Absorbance = 2 – log_10_ (%*T*)).

Pyrolysis profiles of the polymerized products were collected by a thermogravimetric analyzer (TGA, Q500 TA Instruments) under a nitrogen flow of 90 mL/min from room temperature to 800 °C with a heating rate of 10 °C/min.

Samples were prepared for electron microscopy on copper “C-flat” grids with a carbon support film perforated by holes 2 μm in diameter (Protochips, NL). For each specimen, a drop of ~5 μL was drawn from a well-mixed vial, deposited directly onto a glow-discharged grid, and then dried gently under a warm lamp for 30 min. Energy-dispersive X-ray spectroscopy (EDX) measurements were acquired in a Talos 200-X microscope (Thermo-Fisher Scientific) equipped with a QUANTAX FlatQUAD spectrometer (Bruker) and analyzed using the embedded Velox software. Spectra are presented after automated background subtraction and peak detection.

Scanning transmission electron tomography was performed on a Tecnai T20-F microscope (FEI, Inc.) equipped with a Fischione model 3500 high-angle annular dark-field detector. The data set consisted of 41 tilt images acquired in dose-symmetric mode covering a range from −60° to 60° with an interval of 3°. The tilt series was aligned by the “patch tracking” method and reconstructed using the etomo program in IMOD^20^ and displayed using UCSF Chimera.^21^

## 3 Results and Discussion

### 3.1 Formation of Silica–Lignin and Silica Gel

Coniferyl alcohol was polymerized by HRP and H_2_O_2_ activity to produce a dehydrogenated polymer. An opaque solution formed within seconds and indicated the fast formation of a lignin-like polymer. About 65% of the coniferyl alcohol polymerized into synthetic lignin, as determined by weighing the reaction product. Adding 5, 10, or 20 mM silicic acid to the polymerization reaction did not increase its yield when sampled after 3 h. This indicated that the supersaturated silicic acid solution did not have time to polymerize (Table 1). Note that supersaturated silicic acid solution may be stable for several hours.^22^ However, adding 5 or 10 mM silicic acid and allowing the reaction to proceed for 3 days increased the total yield (lignin + silica) by 20–50% compared to the polymerization product of coniferyl alcohol. Adding 20 mM silicic acid increased the total yield threefold. This suggested autopolymerization of silicic acid at a concentration 10 times higher than its equilibrium solubility, which in pure water is 1.7 mM.

**Table 1:** Weight of Dried Synthetic Lignin and Lignin–Silica Polymerization Products^a^^,^^b^

initial Si(OH)_4_ (mM)	maximal SiO_2_^c^ (mg)	product at 3 h (mg)	product at 3 d (mg)			TGA-calculated lignin yield^d^ (mg)		
0	0	1.9 ± 0.1	2.0 ± 0.1					
		**LSi-S**	**LSi-S**	**LSi-L**	**LSi-WL**	**LSi-S**	**LSi-L**	**LSi-WL**
5	1.21	2.0 ± 0.1	2.3 ± 0.2	2.2 ± 0.2	2.5 ± 0.2	UD	UD	UD
10	2.43	2.0 ± 0.1	2.9 ± 0.2	3.1 ± 0.2	3.1 ± 0.2	UD	UD	UD
20	4.86	2.0 ± 0.1	5.6 ± 0.2	5.9 ± 0.2	6.4 ± 0.2	2.2 ± 0.1	2.2 ± 0.1	2.0 ± 0.1
50	12.15	9.8 ± 0.2	12.8 ± 0.2	13.6 ± 0.2	14.1 ± 0.2	2.3 ± 0.1	2.2 ± 0.1	1.9 ± 0.1

a Initial weight of coniferyl alcohol was ∼3 mg. Reported average weight ± standard deviations are of at least three measurements.

b LSi-S, lignin polymerized in the presence silicic acid; LSi-L, silicic acid added to polymerizing lignin after most of the reaction had finished; LSi-WL, silicic acid added to polymerized lignin with removal of lignin precursors. UD, undetermined. Weight based on TGA weight loss could not be determined because lignin pyrolysis formed char.

c Theoretical weight of SiO_2_ with respect to initial Si(OH)_4_ concentration.

d Calculated weights of lignin in lignin–silica polymers based on weight loss from TGA and initial dried product weight. Weight based on TGA weight loss could be determined only when lignin pyrolysis led to evaporation with no formation of char.

**Figure 2:**
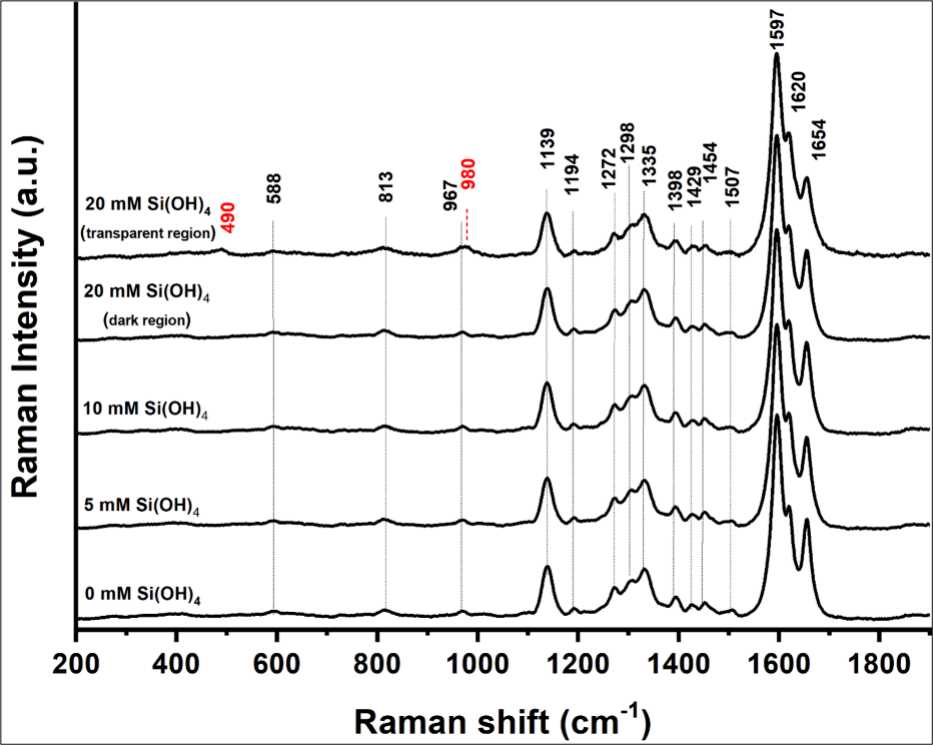
Raman spectra of coniferyl alcohol oxidative coupling without and with silicic acid reacted for 3 days. Bands marked in black text are assigned to synthetic lignin. Bands marked in red text are assigned to silica gel (Table 2) and appear only in transparent regions (Figure 1S). Relative intensity of the band 1654 cm^–1^ in relation to 1597 cm^–1^ is 0.49 for 0 mM Si(OH)_4_, 0.45 for 5 mM Si(OH)_4_, 0.45 for 10 mM Si(OH)_4_, 0.44 for 20 mM Si(OH)_4_ (dark region), and 0.39 for 20 mM Si(OH)_4_ (transparent region).

Raman microspectroscopy identified two forms of product within the samples prepared for 3 days (Figure 2). Dark regions were characterized by lignin-like bands that appeared similarly in samples prepared with and without silicic acid. Transparent regions were identified in samples prepared with 20 mM and higher concentrations of silicic acid (Figure S1). These regions exhibited a broad band around 490 cm^–1^ assigned to amorphous silica, and a band at 980 cm^–1^ assigned to symmetric stretching vibrations of silanol groups. Additional Raman bands of HRP were not detected even in unwashed lignin, indicating that HRP concentration was too low to detect by protein-specific bands (Table 2, Figure S2).

**Table 2:** Assignment of Bands in Raman Spectra of the Coniferyl Alcohol Polymerization Product and Silica Gel

peak position (cm^–1^)	assignment	ref
1700	C=O stretching of para benzo quinone methide	(23)
1654	ring-conjugated C=C stretch of coniferyl alcohol; C=O stretch of coniferyl aldehyde	(24)
1620	ring-conjugated C=C stretch of coniferyl aldehyde	
1597	symmetric aryl ring stretching	
1570	phenoxyl radical/lignin radicals	(25)
1552	phenoxyl radical/lignin radicals	
1507	asymmetric aryl ring stretching	(24)
1505	phenoxyl radical/lignin radicals	(25)
1454	CH bending of OCH_3_ and CH_2_	(24,26,27)
1429	CH bending of OCH_3_ and CH_2_; C=C stretch of ring	
1398	C–H bend of coniferyl aldehyde	
1335	C–H bending of C=C	
1298	C–H bend of C=C of coniferyl alcohol	
1272	C–H bend of C=C; C=C bend of ring	
1194	aryl–O-H bend, CH_3_ rocking	
1139	C–C stretch of coniferyl aldehyde	
1090	C–H bend/CC stretching	(28)
1076	PO_4_^3–^ ν_3_ antisymmetric stretching mode	(29)
991	CCC bend	(28)
980	symmetric stretching vibrations of silanol groups	(30)
966	PO_4_^3–^ ν_1_ symmetric stretching mode	(29)
967	C–H deformation of C=C	(26)
813	skeletal deformation of aromatic rings, substituent groups, and side chains	(24)
801	ring breath/CCC bend	(28)
588	skeletal deformation of aromatic rings, substituent groups, and side chains	(24)
536	PO_4_^3–^ ν_4_ bending modes	(29)
490	stretching vibration of oxygen atoms symmetrically bonded to Si–OH	(30)
406	PO_4_^3–^ ν_2_ bending modes	(29)

Correlated with the addition of silica, we detected a reduction in the relative intensity of the band at 1654 cm^–1^ (aromatic-ring-conjugated C=C stretch of coniferyl alcohol and C=O stretching of coniferyl aldehyde) with respect to 1597 cm^–1^ (aryl ring stretching in lignin-like polymer, Table 2). Band height ratio reduced from 0.5, in samples prepared with 0–10 mM silicic acid, to 0.4 in samples prepared with 20 mM. This indicated that silica formation decreased the fraction of conjugated C=C and C=O bonds in the synthetic lignin. Extended conjugation forms only in end-groups of β-O-4 and β-5 linkages (Figure 1), suggesting that their fraction decreased in the presence of silicic acid.

### 3.2 Si–O–C Bonds Form During Lignin–Silica Copolymerization

**Figure 3:**
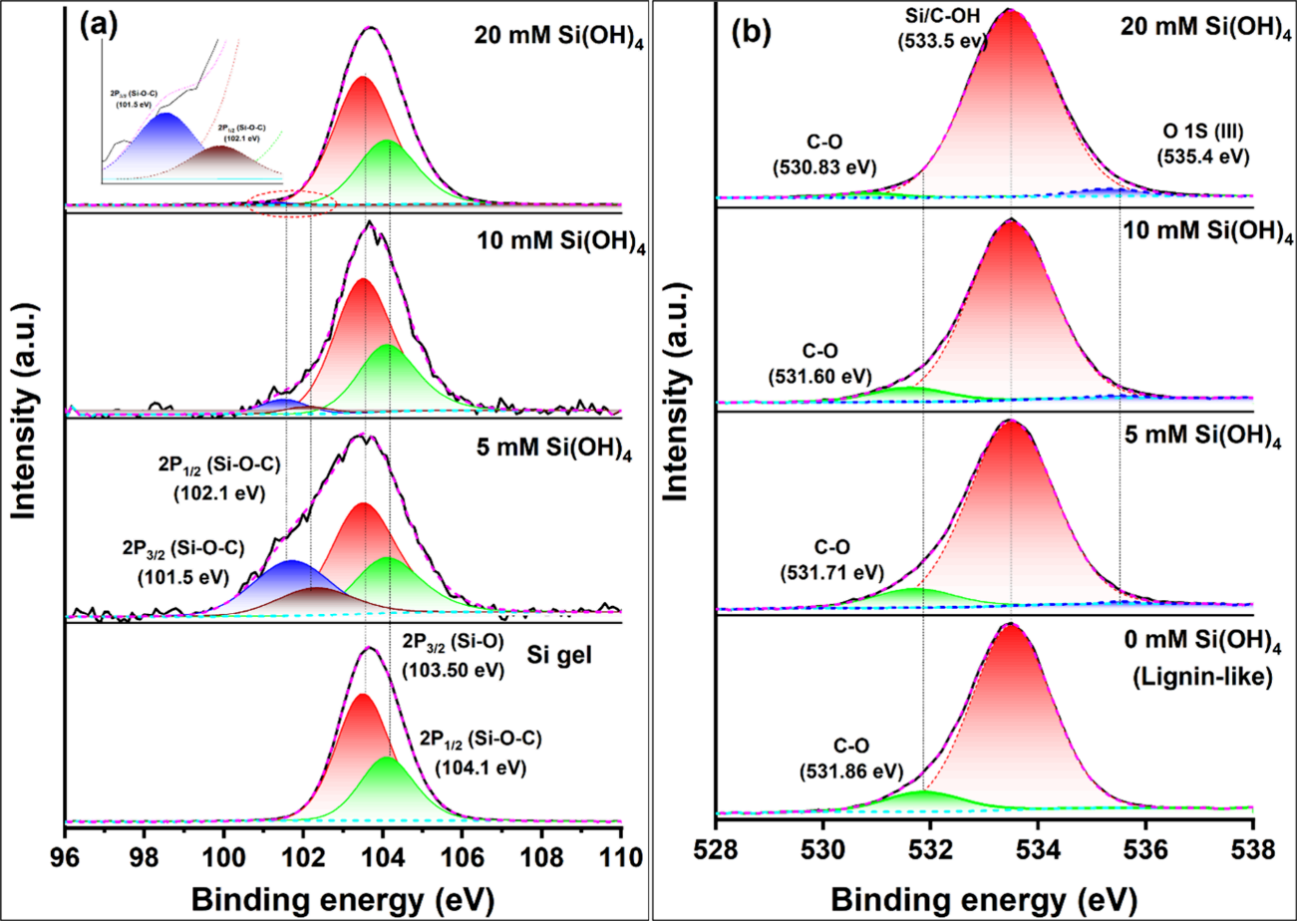
Si 2P and O 1S X-ray photoelectron spectroscopy (XPS) of samples reacted for 3 days. (a) Si 2P level of lignin–silica copolymerized products (three upper panels) and silica gel (lower panel). Black curves indicate the measured intensity. Red curves indicate the sum of all fitted peaks: green, Si–OH; red, Si–OSi; brown and blue, Si–OC. (b) O 1S levels of pure lignin-like polymer (lower panel) and lignin–silica copolymerized products (three upper panels). Black curves indicate the measured intensity. Red curves indicate the sum of all fitted peaks: blue, O–H_2_ of adsorbed surface water in silica-containing samples, red, C–OH, and green, C–OC and C–OSi. Dashed vertical lines indicate shifts in C–O bonds relative to the pure lignin-like polymer.

To test whether Si–O–C formed between silica and the lignin-like polymer, we performed high-resolution X-ray photoelectron spectroscopy (XPS) analyses. Figure 3a compares Si 2p electron orbital spectra of lignin–silica copolymerized products to pure autopolymerized silica gel. The broad peaks covering 103.5 eV (Si 2p_3/2_) and 104.1 eV (Si 2p_1/2_) are attributed to various Si–Ox species such as Si–O and Si–OH, respectively. Lignin–silica products presented, in addition, a doublet peak at 101.5–102.1 eV, indicating a Si–O–C linkage.^31,32^ The relative amount of Si–O–C bonds reduced with increasing silicic acid concentration and an increased fraction of Si–Ox species.

XPS O 1s spectra exhibited a peak centered at 533.5 eV that was assigned to a hydroxyl group bound to carbon or silicon (Figure 3b).^33−35^ A small peak at 535.4 eV was increased with addition of silicic acid and was attributed to absorbed surface water.^36^ Another small peak at 531.86 eV was attributed to C–O–C.^37^ In the presence of silicic acid, this peak was shifted to lower binding energy, and its relative area was decreased compared to that of the pure lignin-like polymer. The reduction in binding energy could be a result of the formation of Si–O–C bonds that are weaker due to the lower electronegativity of Si in relation to C.^31^ The reduction in peak intensity may indicate a lower abundance of C–O–C ether linkages in the lignin–silica copolymers, possibly indicating that the lignin-like units become shorter with the increased silicic acid.^38^

### 3.3 Formation of Lignin Affects Silica Properties

To determine whether coniferyl alcohol monomers, the polymerizing radicals, or the polymerized lignin-like polymer formed Si–O–C bonds, we reacted coniferyl alcohol and silicic acid under three conditions: (1) Silicic acid was added to the reaction solution simultaneously with polymerizing coniferyl alcohol (LSi-S; lignin–silica-simultaneous). (2) Silicic acid was added 3 h after initiation of coniferyl alcohol polymerization (LSi-L; L, late), i.e., after the lignin polymerization had been completed. (3) Silicic acid was added to the polymerized and rinsed lignin-like polymer (LSi-WL; WL, washed late), ensuring the removal of lignin-like precursors.

When 5 or 10 mM silicic acid was added to produce LSi-S, LSi-L, and LSi-WL over 3 days, the solid yields by weight increased by up to 50% compared with lignin–silica synthesized over 3 h. This indicated some polymerization of silica in addition to lignin in contrast to the 3 h samples (Table 1). When silicic acid concentrations of 20 mM and higher were added to the reactions, the yield was close to the combined weight of lignin plus silica. Specifically in LSi-WL samples, the polymerization of silicic acid was maximal. The lower yields in samples LSi-S and LSi-L prepared with 20 and 50 mM Si(OH)_4_ could suggest that silica polymerization was reduced under these conditions.

**Figure 4:**
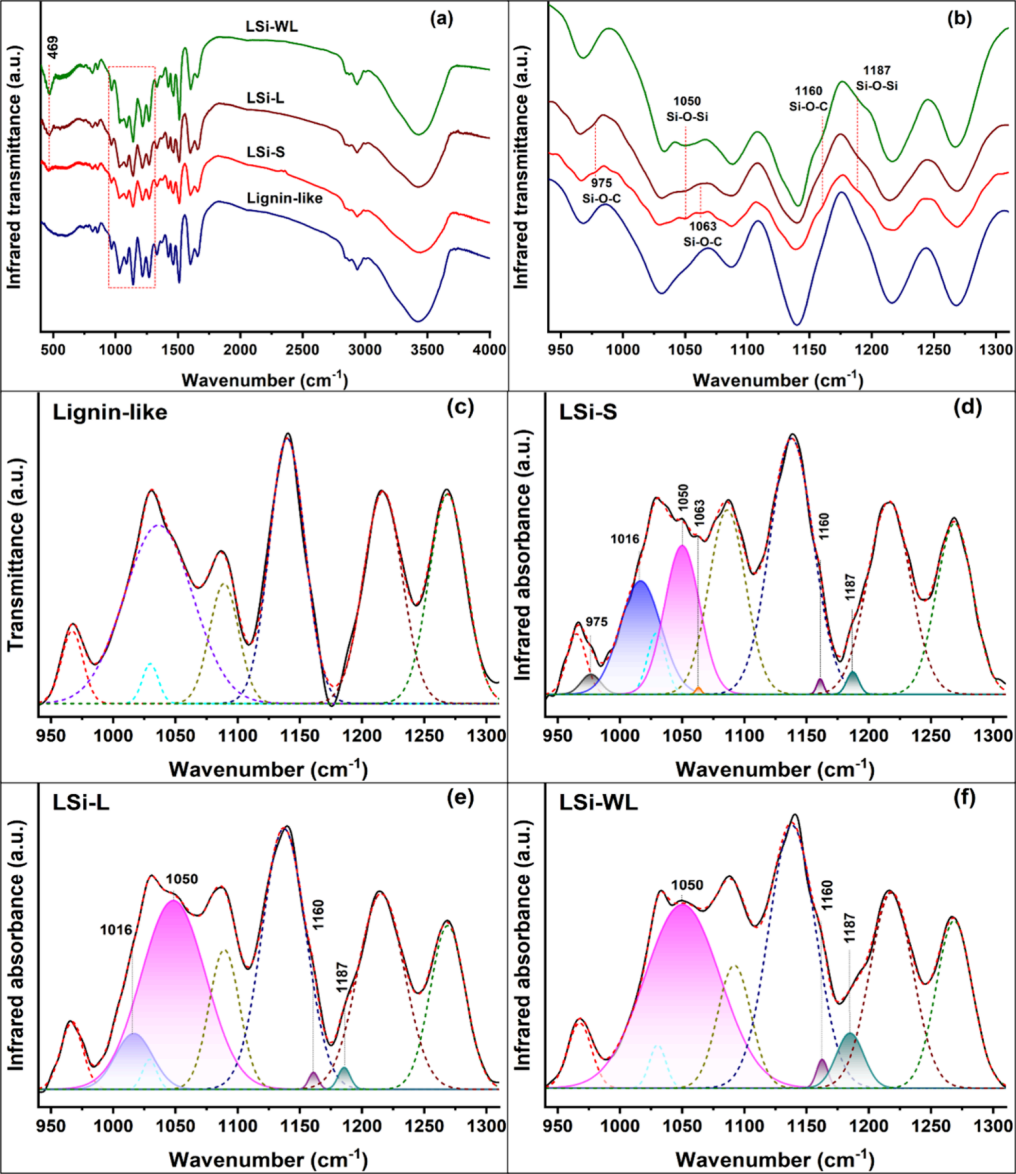
FTIR spectra of synthetic lignin and lignin–silica precipitants. (a) Transmission spectra of lignin and lignin–silica products showing silanol absorption (469 cm^–1^), most prominently when silicic acid was added after lignin polymerization (LSi-WL). The range from 940 to 1310 cm^–1^, bordered by a rectangle, presents the absorptions of Si–O–Si and Si–O–C vibration modes marked in panel b. (b) Transmission spectra of lignin and lignin–silica in the wavenumber range of 940 to 1310 cm^–1^, showing shoulder peaks of Si–O–Si and Si–O–C. (c–f) Deconvolution of the same spectra in absorption mode reveals Si–O–Si and Si–O–C bands on the background of the lignin spectrum. (c) Pure lignin-like polymer (control), (d) LSi-S (lignin polymerized in the presence of silicic acid), (e) LSi-L (silicic acid added to polymerizing lignin after most of the reaction had finished), and (f) LSi-WL (silicic acid added to polymerized lignin with the removal of lignin precursors). Black line represents infrared absorption, and red dashed line represents the sum of deconvoluted peaks of lignin (colored-dashed lines) and Si–O (full lines with filled area). See the text and Table 3 for peak assignments.

Infrared spectra were compared between the lignin-like polymer and lignin–silica LSi-S, LSi-L and LSi-WL products obtained with 10 mM silicic acid (Figure 4, Table 3). A band at 469 cm^–1^, assigned to bending vibrations of Si–O–Si, could be detected in all of the lignin–silica products and was most prominent in LSi-WL (Figure 4a). In the range of 940–1310 cm^–1^, the broad lignin band centered around 1035 cm^–1^ was assigned to the C–O deformation in primary alcohols. This band was transformed into silica-related bands and peak shoulders in the LSi samples (Figure 4b). Deconvolution of the spectra allowed us to resolve six peaks related to silica or Si–O–C vibrations (Figure 4c–f, Table 3). In all LSi samples, we resolved a broad band at 1050 cm^–1^ and a shoulder at 1187 cm^–1^, assigned to Si–O–Si asymmetric stretching of the silica gel. The relative area of these bands was higher in LSi-WL compared to that in LSi-S and LSi-L (Figure 4d–f). All three bands at 469, 1050, and 1187 cm^–1^ indicated that the polymerization of silicic acid was more intense in LSi-WL than in LSi-S and LSi-L. In contrast, a broad band at 1016 cm^–1^ was resolved in LSi-S and LSi-L but not in LSi-WL, and its relative area was greater in LSi-S compared to LSi-L. Its assignment to Si–O–Si asymmetric stretching of cyclic siloxanes (cyclic trimer, Table 3) indicated the existence of partially polymerized silica in LSi-S and LSi-L. Small shoulders at 975, 1063, and 1160 cm^–1^ indicated the formation of Si–O–C bonds: Si–O–C asymmetric stretch attributed to Si-propoxy at 1160 cm^–1^ could be detected in all lignin–silica compounds; the Si–O–C asymmetric stretch attributed to the Si-phenoxy group at 975 cm^–1^ and the C–O–Si ring-link structure at 1063 cm^–1^ could be detected only in LSi-S (Figure 4d, Table 3). This may suggest that silicic acid could attach to the radicalized monomer and bind to it at the O4 position only during the first 3 h of lignin polymerization.

**Table 3:** Assignment of Bands in the Infrared Spectrum of the Coniferyl Alcohol Polymerization Product without and with the Addition of Silicic Acid

observed bands (cm^–1^)	assignment	ref
∼3420	O–H stretching	(27)
∼3475	stretching vibrations of Si–OH groups	(39)
3065	C–H stretching of aromatic ring	(27)
3002	C–H stretch of OCH_3_	
2937	symmetric C–H stretch of OCH_3_	
	antisymmetric stretch of CH_2_OH	
2875	symmetric stretch of CH_2_OH	
2844	symmetric C–H stretch of OCH_3_	
1660	C=O stretch of conjugated carbonyls	
	C=C stretching of coniferyl alcohol	
1645	O–H vibrations of physically adsorbed water	(39)
1599	ring stretch of G rings	(27,40)
1507	ring stretch of G rings	
1463	C–H bending of OCH_3_ and CH_2_	
1423	ring stretch of G rings	
1369	ring stretch of 4-OH G rings; aliphatic C–H stretch in CH_3_, not in OMe	
1330	ring stretch of asymmetric-tetrasubstituted rings	
1267	ring bend of G rings	
1213	ring bend of G rings	
1200	longitudinal optical modes of Si–O–Si asymmetric stretching vibrations	(39)
1187	longitudinal optical modes of Si–O–Si asymmetric stretching vibrations	
1160	Si–O–C asymmetric stretching or Si-propoxy	(41,42)
1141	aromatic C–H in-plane deformation; secondary alcohols; C–O stretch	(27)
1135	Si–O–Si stretching vibration of and ladder-like polymers	(43)
1124	Si–O–Si stretching vibration of cage-like polymer	
1098	transversal optical modes of Si–O–Si asymmetric stretching vibrations	(39)
1087	C–O stretch in secondary alcohols and aliphatic ethers	(27)
1063	Si–O–C asymmetric stretching or C–O–Si ring link structure	(41,42)
1050	Si–O–Si asymmetric stretching of open-hain siloxanes	(42)
1029–1035	aromatic C–H in-plane deformation; C–O deformation in primary alcohols; C–O stretch (unconjugated)	(27)
1016	Si–O–Si asymmetric stretching of cyclic siloxanes (cyclic trimer)	(42)
975	Si-phenoxy	(41)
967	HC=CH out-of-plane deformation	(27)
960	Si–O in-plane stretching vibrations of the silanol Si–OH	(39)
930	aromatic ring C–H out-of-plane deformation	(27)
856	C–H out-of-plane in positions 2, 5, and 6 of G-units	
820	C–H out-of-plane in positions 2, 5, and 6 of G-units	
800	Si–O–Si symmetric stretching vibrations	(39)
783	ring bend of G rings	(27)
737	ring bend of G rings	
604	cyclic (Si–O)_4_ moiety	(43,44)
469	Si–O–Si bending vibrations	(39)

**Figure 5:**
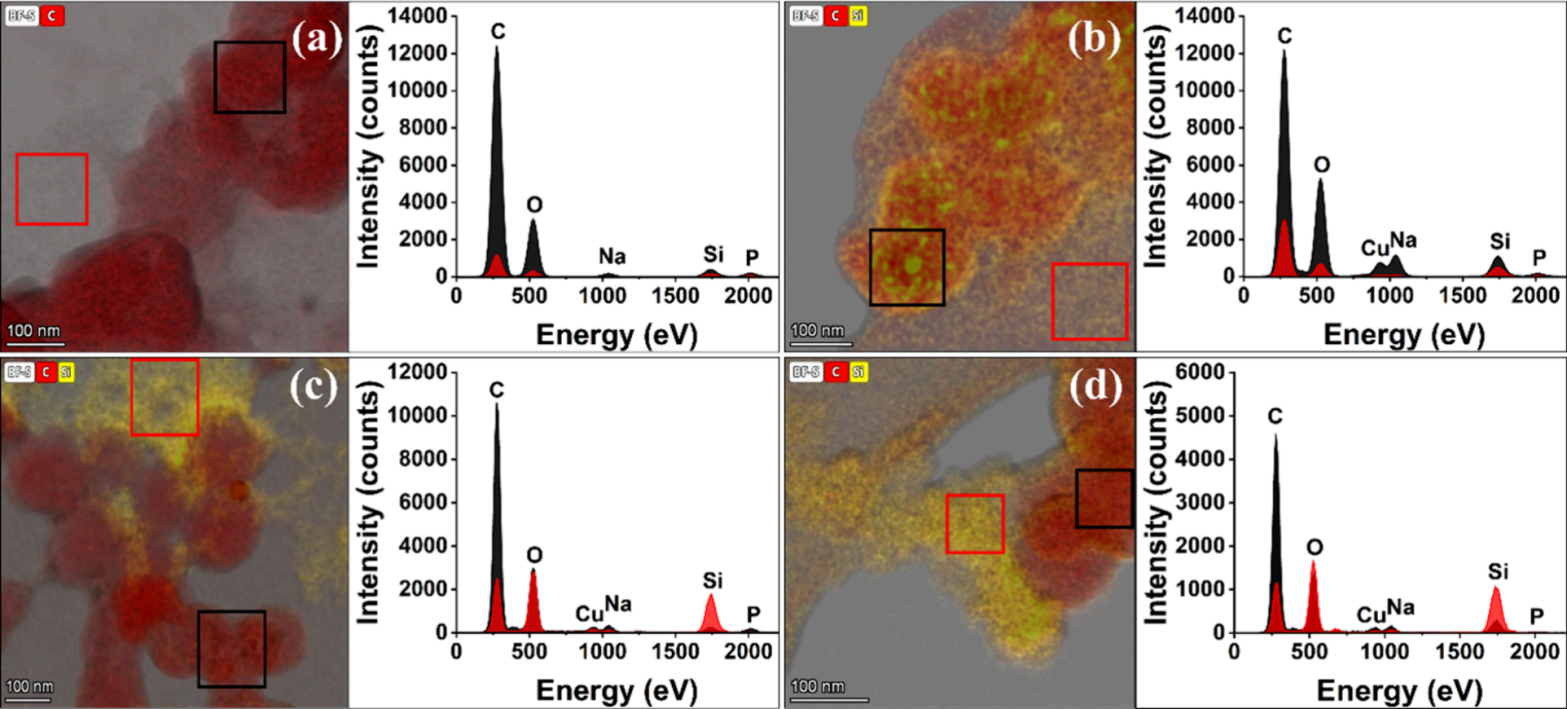
Scanning transmission electron microscopy of synthetic lignin and lignin–silica samples (a) Left: synthetic lignin sample shows particles of ∼100 nm that form a network. Energy-dispersive X-ray spectroscopy (EDX) analysis indicates a minute background of Si signals in yellow. Right: EDX spectra collected from the particles (black) and the background (red) also show minute Si content. (b) In LSi-S, lignin polymerized in the presence of silicic acid, we identified a similar morphology of the synthetic lignin particles. Left: EDX imaging of Si (yellow) indicates silica formation in or on the particles (right, black spectrum) more than between the particles (red spectrum). (c) In LSi-L, silicic acid was added to polymerizing lignin after most of the reaction was completed. Left: EDX Si distribution (yellow) indicated both silica in association with the lignin particles and between particles, with a gel extending away from them. Right: EDX spectra were collected from the highly concentrated Si region deposited as silica gel (red) and nonsilicified lignin particles (black). (d) In LSi-WL, silicic acid was added to polymerized lignin with the removal of lignin precursors. Left, EDX Si distribution (yellow) indicated silica gel (right, red spectrum) between the lignin particles that were less silicified (black spectrum). Regions of interest in the left panels are marked with color matching the spectral traces in the right panels.

Scanning transmission electron microscopy (STEM) of a pure lignin-like polymer revealed a network of sphere-like particles of about 100 nm. The lignin–silica samples showed a similar organic network with a more condensed and less homogeneous structure in comparison to the lignin-like polymer. The precipitation of 2–5 nm silica particles into the lignin-like polymer network was confirmed by energy-dispersive X-ray spectroscopy (EDS). In LSi-S and LSi-L, we could find silica particles embedded in the spherical organic particles. In LSi-L and LSi-WL, we identified nonsilicified lignin particles in the vicinity of silica gel (Figure 5). These results support the interpretation of FTIR that in LSi-S and LSi-L precipitates, the polymer has a significantly different structure compared to the pure lignin-like polymer.

Collectively, the presented results indicated that the lignin-like polymer formed quickly, completing its polymerization within 3 h. Silica, on the other hand, polymerized slowly over 3 days. In the presence of prepolymerized lignin (most prominently in LSi-WL), silicic acid polymerized into a network of silica gel, and Si–O–C bonds formed to the aliphatic hydroxyl/carbonyl groups of lignin. By contrast, in LSi-S and LSi-L samples, the silicic acid was present together with oxidized lignin precursors that affected the structure of the silica precipitate. Most prominently in LSi-S, silicic acid formed small cyclic oligomers with Si-phenoxy bonds and ring-linked Si–O–C bonds.

### 3.4 Formation of Silica Affects Lignin Properties

When Si binds to the phenoxy radicals and/or quinone methide of the dehydrodimers, it may inhibit the lignin C–O–C ether interunit linkages, which would interrupt their polymerization into larger lignin fragments. Such activity is consistent with XPS results, and also with published data showing that during lignin-like polymer synthesis, silicic acid binding to phenylpropanoid dimers slows down their polymerization into larger fragments, resulting in an altered polymer structure.^38^

**Figure 6:**
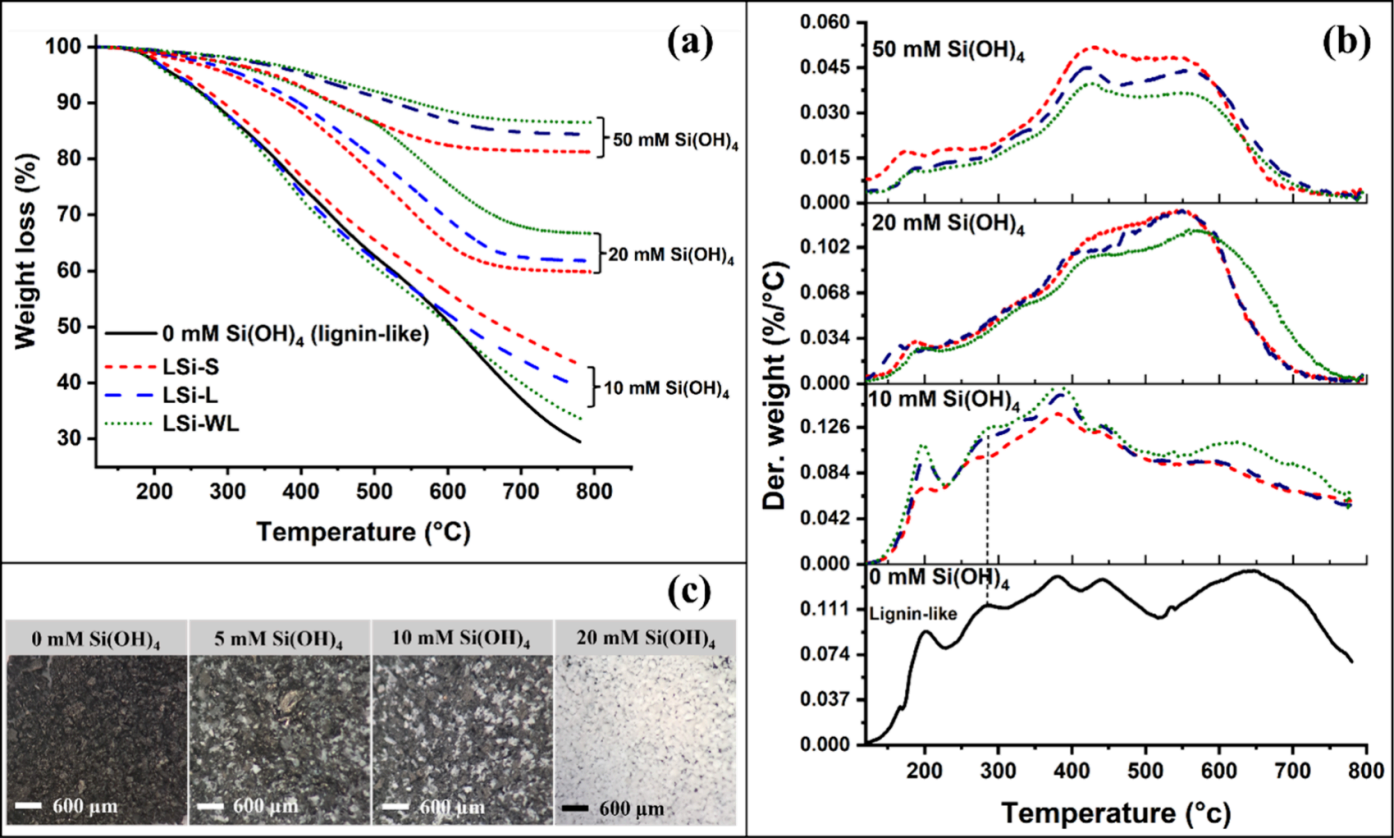
Thermal gravimetric analysis (TGA) of synthetic lignin and lignin–silica samples. (a) Weight loss traces of lignin-like (continuous line) and lignin–silica samples (dashed lines). (b) Weight loss rates (differential thermal gravimetry, DTG) of lignin (continuous black line) and lignin–silica samples (dashed lines). Red: LSi-S (lignin polymerized in the presence of silicic acid), blue: LSi-L (silicic acid added to polymerizing lignin after most of the reaction had finished), green: LSi-WL (silicic acid added to polymerized lignin with the removal of lignin precursors). (c) Images of the residual products after pyrolysis.

Thermogravimetric analyses (TGA) supported structural changes in lignin as a result of lignin–silica interactions (Figure 6). To avoid the influence of loosely bound water molecules, TGA graphs were normalized at 120 °C. The weight loss was divided into primary (120–400 °C) and secondary (400–800 °C) pyrolysis processes.^45^ In primary pyrolysis of lignin-like polymers, the major weight loss is associated with cleavage of interunit linkages, releasing monomeric phenols and their derivatives into the vapor phase. The monomers further degrade during a secondary pyrolysis reaction stage (>400 °C).^46^ In lignin–silica compounds synthesized with a silicic acid concentration ≤10 mM, the weight loss was more than 55%, while in samples synthesized with a silicic acid concentration >10 mM, the weight loss was less than 40% (Figure 6a). This is in accordance with the expected high silica yield in the latter samples (Table 1). We noted that the weight loss of lignin-like samples produced with [Si(OH)_4_] ≤ 10 mM was continuous with temperature, while in the samples produced with [Si(OH)_4_] > 10 mM, minimal weight loss was detected above 650 °C.

The maximal rates of weight loss, as highlighted by differential thermal gravimetry (DTG), indicated that the synthetic lignin and LSi samples made with [Si(OH)_4_] ≤ 10 mM degraded similarly in the primary stage of pyrolysis (at 200, 283, 340, and 382 °C). However, the decomposition peak at ~ 283 °C, associated with cleavage of alkyl-aryl ether (β-O-4) interunit linkages, was shifted to a lower temperature (∼263 °C) in LSi-S (Figure 6b). This shift suggests a lower concentration of β-O-4 linkages and shorter lignin-like chains in LSi-S copolymer as compared to the pure lignin-like polymer,^47,48^ in agreement with a higher fraction of Si-phenoxy bond indicated by IR spectroscopy (Figure 4d) and published literature.^38^ Samples prepared with [Si(OH)_4_] > 10 mM showed two broad decomposition peaks at around 200 and 315 °C, likely due to the decomposition of lignin into its monomers and striping of hydroxyl groups from SiO_2_ surface.

The product of the primary pyrolysis reaction is mostly phenyl propanoid radicals. During the secondary pyrolysis, these may repolymerize into char or evaporate if stabilized by an H-donor.^45^ In the secondary pyrolysis stage, the lignin-like samples decomposed at a relatively high rate, as shown by two hump-like DTG peaks at ∼640 and ∼715 °C (Figure 6b). Such decomposition behavior usually produces a high proportion of residual carbon.^48^ Interestingly, only a small proportion of residual carbon formed in the LSi samples prepared with 10 mM Si(OH)_4_, and almost no char formed in LSi samples prepared with 20 and 50 mM Si(OH)_4_ (Figure 6c). This suggested that during pyrolysis, SiO_2_ stabilized phenyl propanoid radicals, leading to their gasification instead of charring. All lignin–silica samples showed a broad decomposition peak in the 550–600 °C range, possibly due to gasification of catechols (1,2-dihydroxybenzenes).^45^

Since lignin in the presence of SiO_2_ evaporated without char formation, we could calculate its fraction in the samples, based on percentage weight loss in the temperature range of 200–650 °C (Table 1). Interestingly, the highest yield of lignin-like polymer was obtained when polymerization occurred in the presence of silicic acid, in LSi-S samples, and the lowest yield was obtained in LSi-WL samples, where lignin polymerized without silicic acid. This is evidence that silicic acid promotes lignin polymerization.

**Figure 7:**
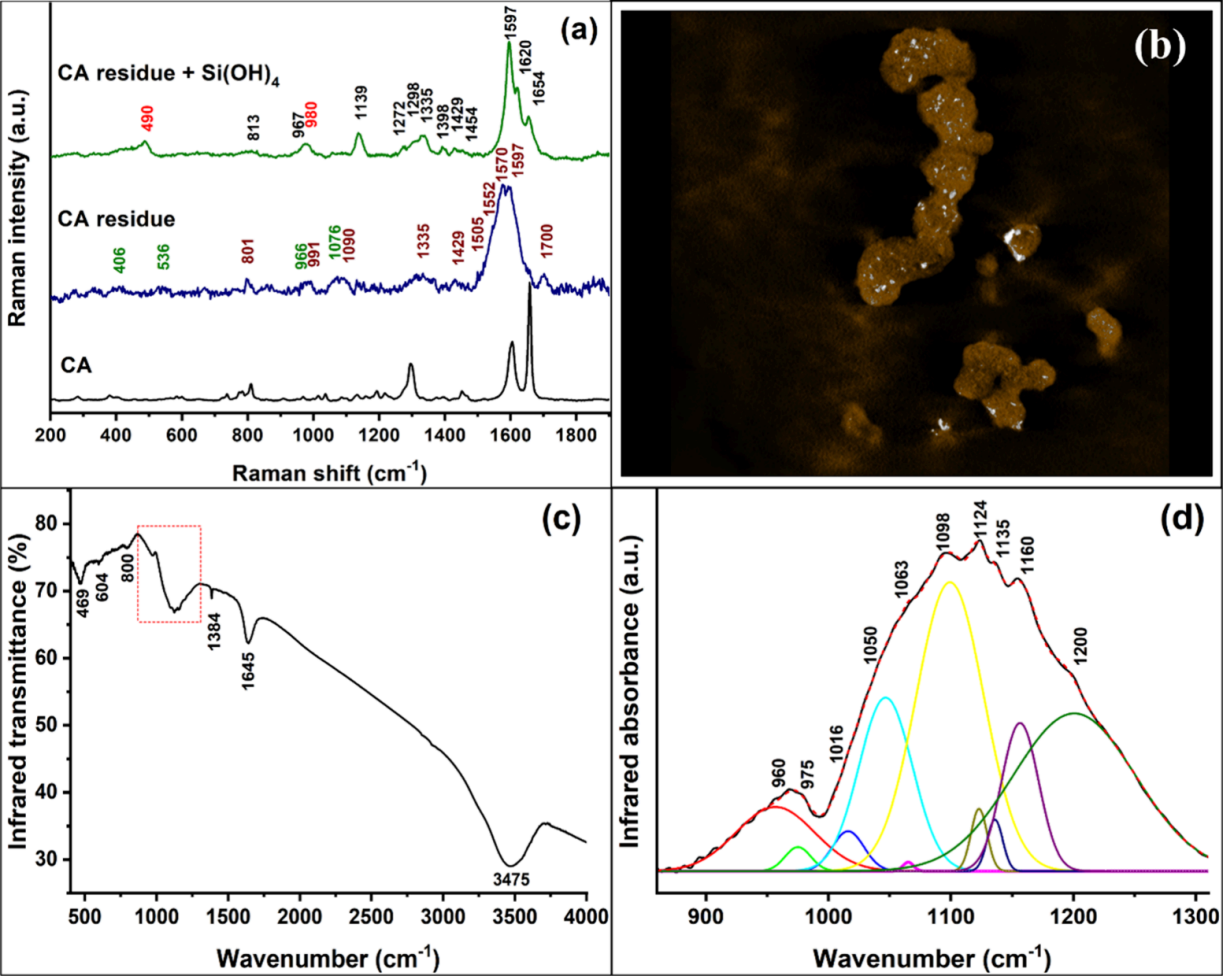
Spectroscopy and electron microscopy of the residue from lignin synthesis, supplemented with silicic acid. (a) Raman spectra of coniferyl alcohol (CA), the residue from the lignin synthesis (CA residue), and the sediment of the reaction of silicic acid with the residue of the lignin synthesis (CA residue + Si(OH)_4_). Text in wine marks radical bands; in green, phosphate ions; in black, lignin-like bands; and in red, silica gel bands (see also Table 2). (b) A slice from a 3-D reconstructed HAADF-STEM tomography model (Video Clip S1), which is based on electron scattering and is highly sensitive to the atomic number of the elements in the sample.^51^ Therefore, silica appears with high contrast against the background of organic lignin. Accordingly, the tomogram was segmented for display such that high-intensity scattering appears in saturated white, while lower intensities appear in semitransparent brown. Silica particles of 2–5 nm are shown embedded within the lignin spherical particles. Field of view is 1.58 μm. (c) FTIR transmission spectra of silicic acid polymerized with coniferyl alcohol radicals, showing cyclic (Si–O)_4_ moieties (604 cm^–1^) in addition to Si–O–Si bands (Table 3). The range from 860 to 1310 cm^–1^, marked by a rectangle, represents Si–O–Si and Si–O–C bonding modes analyzed in panel d. (d) Deconvolution of the Si–O–C bonds, and network, cyclic, and cage-like Si–O–Si FTIR absorption bands (see Table 3 for assignments).

To test whether silicic acid interacts with residues of the lignin polymerization reaction rather than fresh coniferyl alcohol, the supernatant of the lignin polymerization reaction was collected 3 h after its initiation. Raman spectra of a dried droplet of the supernatant revealed the presence of phenoxyl/quinone methide radicals and phosphate ions (Figure 7a, Table 2). When silicic acid (10 mM) was added to the supernatant solution and agitated for 24 h, we collected a sediment that showed a typical lignin-like Raman spectrum, along with silica-related bands at 490 and 980 cm^–1^ (Figure 7a). Electron imaging of the sediment identified spherical particles identical to the lignin-like particles. A 3-D tomographic reconstruction of a STEM tilt series revealed embedded silica particles of about 2–5 nm diameter (Figure 7b) as well as a crust of silica coating some of the lignin-like spheres (Video Clip S1).

According to the widely accepted model originally proposed by Freudenberg and Neish,^49^ β-O-4 linkages occur via coupling of resonance-stabilized phenolic radicals with the intermediate quinone methide. β-ether quinone methides are then aromatized by the nucleophilic addition of a water molecule (Figure 1). This reaction is slow in the absence of an acid catalyst.^50^ Silicic acid may compete with H_2_O and stabilize intermediate quinone methides to precipitate short lignol fragments.

FTIR spectra of the sediment that formed when silicic acid reacted with the residual radicals supported the formation of silica gel and lignin and indicated Si–O–C bonding (Figure 7c,d and Table 3). In addition to Si–O–Si network bands, IR spectra exhibited a band at 1016 cm^–1^ associated with strained cyclic siloxanes and cyclic-organo-bridged silsesquioxanes. A specific band at ∼604 cm^–1^ was observed, assigned to cyclic (Si–O)_4_ moieties (Figure 7c and Table 3). Two sharp bands appeared at 1124 and 1135 cm^–1^, corresponding to the Si–O–Si stretching vibration of a cage-like arrangement that contained phenyl units.^43^ A sharp band at 1384 cm^–1^ was assigned to the C–H symmetric deformation of −CH_2_ of alkoxy groups.^39^ Furthermore, we could resolve various Si–O–C bonds at 975, 1063, and 1160 cm^–1^. Interestingly, all these bands were detected during the first 3 h of lignin–silica copolymerization in the LSi-S reaction, where silica gel formation was negligible compared to lignin (Figure S3, Table 3). These results, therefore, confirm that phenoxy radicals and/or quinone methide equilibrate small polysilicic acid species upon reaction with monosilicic acid. Modifications of the structures of silicic acid oligomers by phenoxy radicals reduced the conformational freedom of the siloxane chain, which shifted the equilibrium toward the cyclic and cage arrangement side, resulting in a decrease in silica polymer yield.

## 4 Conclusions

Our results show that lignin functional groups inhibit silica polymerization and, reciprocally, that silicic acid binding to lignin monomers alters the lignin structure. Moreover, silicic acid increased the maximal yield of coniferyl alcohol polymerization by 6–10%. The results suggest that during the initial stage of lignin–silica polymerization, the quick formation of lignin enables its growth to form ∼100 nm diameter particles that bind covalently with oligomeric silicic acid. The C–O–Si(OH)_3_ functional groups serve as a nucleant for 2–5 nm silica particles. Later on, silica gel forms between the particles depending on the concentration of silicic acid in the reaction solution.

Our in vitro data may suggest that during lignin formation in planta, silicic acid could also compete with monolignol phenoxyl radicals on the extension of lignin. This extension could cause a red shift in the fluorescence of nonsilicified lignin, as we found previously in lignin associated with silica in sorghum roots.^52^ Additional work should determine whether the chemical principles we identified are applicable to naturally synthesized plant lignin.

## Supporting Information Available

Optical microscopic images of the synthetic lignin and lignin–silica samples; Raman spectra of horseradish peroxidase (HRP) and unwashed lignin-containing HRP residues; and FTIR spectra of synthetic lignin and LSi-S copolymer synthesized for 3 h (PDF)
Electron tomography of the sample of silicic acid mixed with lignin polymerization supernatant solution (MP4)

## Notes

EDS and tomography datasets are available from zenodo.org with doi: 10.5281/zenodo.11099398.
