# Supplementary material for: Silica Biomineralization with Lignin Involves Si–O–C Bonds That Stabilize Radicals
Source: Biomacromolecules. 2024 May 7;25(6):3409–19. doi: 10.1021/acs.biomac.4c00061 (PMC11170934; doi:10.1021/acs.biomac.4c00061)
Supplement: Supplementary file 1 — bm4c00061_si_001.pdf [file bm4c00061_si_001.pdf]

Silica biomineralization with lignin involves Si-O-C bonds that stabilize radicals

Srinath Palakurthy[1], Lothar Houben[2], Michael Elbaum[2], and Rivka Elbaum[1*]

[1]The Robert H. Smith Institute of Plant Sciences and Genetics in Agriculture, The Hebrew University of Jerusalem, 7610001 Rehovot, Israel

[2]The Weizmann Institute of Science, 7610001 Rehovot, Israel

* Corresponding author: Rivka Elbaum (email id: rivka.elbaum@mail.huji.ac.il)

Supporting information

Fig. S1. Representative optical microscopic images of the synthetic lignin and lignin-silica samples.

Fig. S2. Raman spectra of horseradish peroxidase (HRP) and unwashed lignin containing HRP residues

Fig. S3. FTIR transmission spectra of synthetic lignin and lignin-silica LSi-S copolymer synthesized for three hours.

Video Clip S1. Electron tomography of the sample of silicic acid mixed with lignin polymerization supernatant solution.

Supporting Figure S1

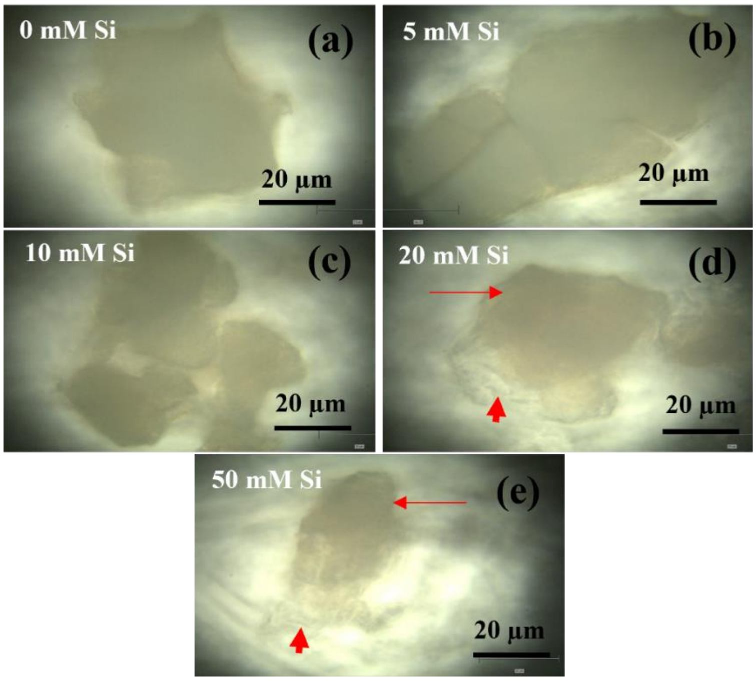

Figure S1: Representative optical microscopic images of the lignin (a) and lignin-silica (b-e) samples. Silicic acid (Si) concentrations are indicated on the panels. Raman measurements at regions marked by long arrows showed bands typical to coniferyl alcohol polymerization product. Raman measurements at regions marked by short arrows showed bands of silica, in addition to coniferyl alcohol polymerization product.

Supporting Figure S2

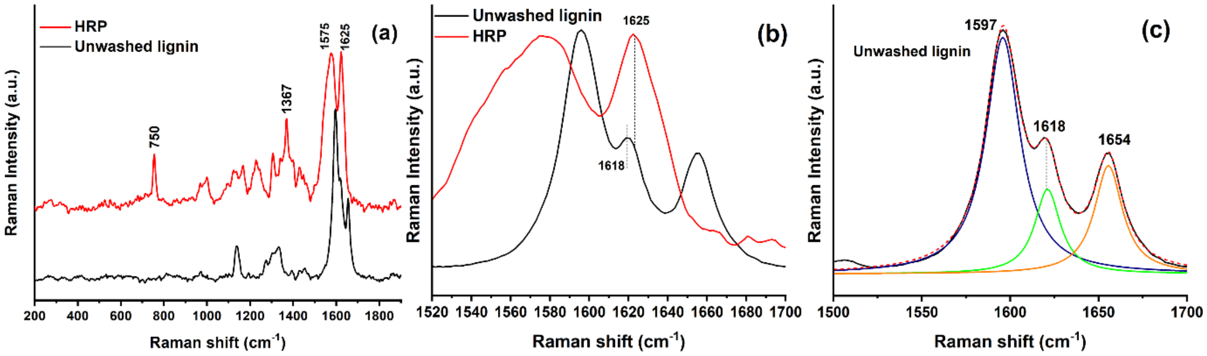

Figure S2: To test for any HRP leftovers in the synthetic lignin, Raman spectra were collected from HRP and compared to unwashed lignin, containing HRP residues. (a) HRP shows intense bands at 750 cm^{-1} of C-S of cysteine residue, and 1367 and 1575 cm^{-1} of CN- and CO-ferrous forms [1,2], missing in the unwashed lignin. (b) A close-up on the typical aromatic bonds showing that the broad 1575 cm^{-1} band of the HRP does appear as a background signal in the unwashed lignin. (c) Fitting the unwashed lignin indicates only 3 bands without any evidence for HRP bands.

Supporting Figure S3

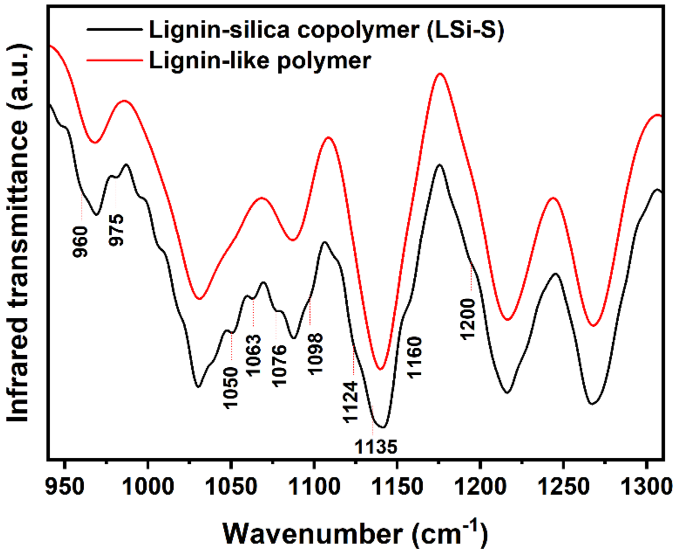

Figure S3. FTIR transmission spectra of synthetic lignin and lignin-silica LSi-S copolymer synthesized for three hours. Sample of polymerized coniferyl alcohol with addition of 20 mM silicic acid (black line) and without silicic acid (red line) were collected 3 h after polymerization was initiated. Lignin and LSi-S show strong lignin peaks. Shoulder peaks of Si-O-Si and Si-O-C in the range from 940 to 1310 cm^{-1} were detected in LSi sample. For peak assignments, see Table 3 in the main text.

Supporting Video Clip S1

Video Clip S1. Electron tomography of the sample of silicic acid mixed with the supernatant solution collected after lignin polymerization. The HAADF-STEM method is based on electron scattering and is highly sensitive to atomic number of the elements in the sample. Therefore, silica appears with high contrast on the background of organic lignin. Accordingly, the tomogram was segmented for display such that high intensity scattering appears in saturated white colour, while lower intensities appear in semi-transparent brown. The video clip shows a rocking representation of the volume projection. Field of view 1.58 µm.

References

(1) Palaniappan, V.; Terner, J. Resonance Raman Spectroscopy of Horseradish Peroxidase Derivatives and Intermediates with Excitation in the near Ultraviolet. *Journal of Biological Chemistry* **1989**, *264* (27), 16046–16053. https://doi.org/10.1016/s0021-9258(18)71585-8.

(2) Wang, B.; Zhang, Y.; Venkitasamy, C.; Wu, B.; Pan, Z.; Ma, H. Effect of Pulsed Light on Activity and Structural Changes of Horseradish Peroxidase. *Food Chem* **2017**, *234*, 20–25. https://doi.org/10.1016/j.foodchem.2017.04.149.
